# Editorial: Probiogenomics of classic and next-generation probiotics

**DOI:** 10.3389/fmicb.2022.982642

**Published:** 2022-08-17

**Authors:** Rodrigo D. O. Carvalho, Eric Guédon, Flávia F. Aburjaile, Vasco Azevedo

**Affiliations:** ^1^Department of Biochemistry and Biophysics, Institute of Health Sciences, Universidade Federal da Bahia, Salvador, Brazil; ^2^STLO, Institut Agro, INRAE, Rennes, France; ^3^Preventive Veterinary Medicine Department, Veterinary School, Universidade Federal de Minas Gerais, Belo Horizonte, Brazil; ^4^Institute of Biological Sciences, Department of Genetics, Ecology, and Evolution, Universidade Federal de Minas Gerais, Belo Horizonte, Brazil

**Keywords:** beneficial microbes, gut microbiota, systems biology, genomics, lactic acid bacteria

Probiotics are microorganisms with the ability to promote human, animal, or plant health. They have been proposed as an alternative approach for treating several diseases, primarily intestinal disorders. Recently, an approach coined as Probiogenomics, based on new powerful sequencing technologies and computational methods, have significantly improved the identification and characterization of probiotic properties by allowing massive amounts of biological data to be screened in a short period of time. Most probiotic strains identified to date belong to the group of lactic acid bacteria. One well-known example of this group are the lactobacilli, used in fermented dairy foods. Recently, however, newly identified taxa with strict ecological association to the host, known as “next-generation probiotics,” are beginning to take the stage. In this context, there are many unknowns to explore concerning the diversity of bacterial strains presenting novel specific traits.

This Research Topic consists of five original articles aiming at identifying novel strains or characterizing already known probiotic formulations available on the market by applying probiogenomics. Some of these studies also integrated *in silico* analysis into exploratory essays. Taken all together, these studies characterize a genome of 58 strains belonging to 14 bacterial species, as well as one yeast presenting potential probiotic properties. Whole-genome analysis was generally suitable for providing more specific resolution for taxonomic classification by applying robust multi-locus phylogeny methods. Similarly, most studies used *in silico* prediction for screening safety-related genetic features such as virulence factors, including hemolysin genes, antimicrobial resistance genes, and mobile elements.

In this latter context, Lugli et al. propose a novel screening method using shotgun metagenomics and flow cytometry to accurately determine the microbe species or contaminants in probiotic formulations. Their study revealed significant inconsistencies regarding taxonomy and composition in several probiotic supplements. Their results might therefore offer an alternative solution for better quality control and could be of use for further studies on developing novel products.

Another advantage of comprehensive genomics in the bioprospection of probiotic strains is the possibility of finding novel unique traits associated with either health promotion or biotechnological applications. Stergiou et al. identified new adhesins and exopolysaccharide genes in *Lactiplantibacillus pentosus* L33 that are involved in host-microbe and microbe-microbe interactions, including adhesion to intestinal cells and formation of microbial biofilms. Similarly, Garcia-Gonzalez et al. used probiogenomics to investigate three *Lactiplantibacillus plantarum* strains, IMC513, C9O4, and LT52, isolated from the human gut, table olives, and raw-milk cheese, respectively. Several bacteriocin genes were predicted in the genome of the three strains, suggesting antimicrobial properties.

As probiotics are living cells, they can respond to specific stimulation. Occasionally, their beneficial effects can be achieved only when they grow under certain circumstances, thereby producing alternative metabolic compounds. Chamberlain et al. performed metabolome analysis to evaluate the impact of a pomegranate extract containing polyphenolic compounds on the metabolic profiles of three lactobacilli strains. Their results showed several metabolites unique to each strain when grown in this way, presenting relevant properties that might contribute to host functions.

Probiogenomics in combination with *in vitro* and *in vivo* essays is considered a more robust approach for discovering and characterizing probiotic strains. For example, Suphoronski et al. identified an unusual beneficial *Enterococcus faecium* strain, LAC7.2, in the gut microbiota of Nile tilapia fishes. This strain demonstrated antimicrobial properties against fish pathogens, including *Francisella* sp. and *Streptococcus* sp., possibly *via* the secretion of enterocin, a bacteriocin predicted in its genome. Moreover, 16S rRNA metagenomic analysis revealed the capacity of LAC7.2 to maintain a balanced gut microbiome composition in a fish model of infection.

These outcomes will certainly have an effect on quality control procedures and hold promise for application in the current or future commercialization of these probiotic strains.

## Author contributions

RC, EG, FA, and VA wrote and edited the manuscript. All authors have contributed substantially to the article and approved the manuscript for publication.

## Conflict of interest

The authors declare that the research was conducted in the absence of any commercial or financial relationships that could be construed as a potential conflict of interest.

## Publisher's note

All claims expressed in this article are solely those of the authors and do not necessarily represent those of their affiliated organizations, or those of the publisher, the editors and the reviewers. Any product that may be evaluated in this article, or claim that may be made by its manufacturer, is not guaranteed or endorsed by the publisher.

